# Increased transferrin saturation is associated with subgingival microbiota dysbiosis and severe periodontitis in genetic haemochromatosis

**DOI:** 10.1038/s41598-018-33813-0

**Published:** 2018-10-19

**Authors:** Emile Boyer, Sandrine Le Gall-David, Bénédicte Martin, Shao Bing Fong, Olivier Loréal, Yves Deugnier, Martine Bonnaure-Mallet, Vincent Meuric

**Affiliations:** 1 Univ Rennes, INSERM, INRA, CHU Rennes, Institut NuMeCan (Nutrition, Metabolism and Cancer), Rennes, F-35000 France; 2 CHU de Rennes, Service d’Odontologie, Rennes, 35033 France; 3 CHU de Rennes, Service des Maladies du Foie, Rennes, 35033 France; 4 CIC 1414, Inserm, Rennes, 35033 France

## Abstract

Genetic haemochromatosis (GH) is responsible for iron overload. Increased transferrin saturation (TSAT) has been associated with severe periodontitis, which is a chronic inflammatory disease affecting tissues surrounding the teeth and is related to dysbiosis of the subgingival microbiota. Because iron is essential for bacterial pathogens, alterations in iron homeostasis can drive dysbiosis. To unravel the relationships between serum iron biomarkers and the subgingival microbiota, we analysed samples from 66 GH patients. The co-occurrence analysis of the microbiota showed very different patterns according to TSAT. Healthy and periopathogenic bacterial clusters were found to compete in patients with normal TSAT (≤45%). However, significant correlations were found between TSAT and the proportions of *Porphyromonas* and *Treponema*, which are two genera that contain well-known periopathogenic species. In patients with high TSAT, the bacterial clusters exhibited no mutual exclusion. Increased iron bioavailability worsened periodontitis and promoted periopathogenic bacteria, such as *Treponema*. The radical changes in host-bacteria relationships and bacterial co-occurrence patterns according to the TSAT level also suggested a shift in the bacterial iron supply from transferrin to NTBI when TSAT exceeded 45%. Taken together, these results indicate that iron bioavailability in biological fluids is part of the equilibrium between the host and its microbiota.

## Introduction

Periodontitis is a chronic inflammatory disease that affects tissues surrounding the teeth. It is strongly associated with the major pathogenic “red complex”, including *Porphyromonas gingivalis*, *Tannerella forsythia* and *Treponema denticola*^1^ and thus is considered an infection. Recent advances in the pathogenesis of periodontal disease have suggested that polymicrobial synergy and microbiota dysbiosis together with a dysregulated immune response can induce inflammation-mediated damage in periodontal tissues^2–4^. Interestingly, currently periodontitis is associated with a growing number of systemic diseases, including cardiovascular diseases, adverse pregnancy outcomes, diabetes^5–7^ and hereditary haemochromatosis^8^.

Genetic haemochromatosis (GH), which is related to the HFE gene p.Cys282Tyr mutation, is the most common form of inherited iron overload disease in European population descendants^9,10^. In plasma, iron is associated with transferrin to increase its bioavailability for cells. Transferrin saturation (TSAT) is the ratio between the total number of iron-binding sites on patient plasma transferrin and the number of binding sites occupied by iron. Normally, TSAT ranges between 20% and 45%. Systemic iron metabolism is controlled by hepcidin, whose expression level is adapted to TSAT to regulate the plasma iron levels. Due to an alteration in the HFE-linked transduction signalling pathway, GH is characterized by a hepcidin-deficiency state. The resulting iron egress from macrophages and enterocytes leads to an increased TSAT level. When TSAT exceeds 45%, non-transferrin-bound iron (NTBI), which is an abnormal biochemical form of iron, occurs in the plasma. NTBI consists mostly of iron associated with citrate or ADP and is involved in oxidative stress through reactive oxygen species generation^11–13^. NTBI especially targets the liver and heart, which explains why the classical form of GH is responsible for hepatic cirrhosis and diabetes^14^. However, currently most GH patients are asymptomatic or present with chronic fatigue, abnormal serum transaminase levels, rheumatism and osteoporosis^15,16^ in the absence of cirrhosis and diabetes. To avoid iron toxicity, cells synthesize ferritin to store excess iron. Consequently, the plasma ferritin levels reflect the tissue iron stores. Standard treatment is based on phlebotomy therapy to first clear out and then avoid reconstitution of iron excess. The primary international guidelines advise that the serum ferritin levels should be lower than 50 *μ*g/L as the gold standard for both initial treatment and maintenance therapy^17–20^.

In a previous study, we investigated the periodontal statuses of GH patients. Unexpectedly, we found that all of the patients had periodontitis. Furthermore, we observed a significant link between the periodontitis severity and increased TSAT; patients with a TSAT level greater than 45% had a five times greater risk of severe periodontitis^8^. Iron overload may strongly impact bacterial behaviour^21^. Indeed, iron is an essential growth factor and can act as a virulence factor for *P*. *gingivalis*, which is considered one of the major keystone pathogens driving dysbiosis in periodontitis^2,22^. In addition, excess iron in the sera has been reported to decrease serum antibacterial activity in GH patients^23^. Furthermore, alteration of the gut microbiota has been reported in *Hfe*^−/−^ mice, with the gut microbiota of the *Hfe*^−/−^ mice significantly distinct from that of the wild-type mice. The authors suggested that genetic modification of iron metabolism could influence the composition of potentially probiotic bacterial species^24^.

Therefore, we hypothesized that the alteration of iron metabolism induced by the HFE C282Y gene mutation could modify and drive the subgingival microbiota towards dysbiosis, which could participate in the periodontitis severity. The aim of this study was to investigate a putative association between the subgingival microbiota and serum iron biomarkers in GH patients from our previous study.

## Results

At the time of the dental examination, 21 patients had normal TSAT (≤45%) and 45 patients had high TSAT (>45%). Among the 45 patients with high TSAT, 19 had serum ferritin levels ≤50 *μ*g/L and 26 had serum ferritin levels >50 *μ*g/L. As shown in Table 1, 33.3% of the patients with normal TSAT presented severe periodontitis *versus* 68.9% of those with high TSAT (*χ*^2^, $p=0.006$). A significant increase in all clinical attachment loss (CAL) measures was observed in the patients with high TSAT. Although not significant, we observed a trend towards increased pocket probing depth (PPD) measures in patients with high TSAT. Moreover, no significant differences in demographic data were found between the patients with normal and high TSAT (Supplementary Table S1).

**Table 1:** Periodontitis measures of patients in accordance with their TSAT levels.

Measures of periodontitis	Transferrin saturation		*p*-value
	Normal (≤45%) (*n* = 21)	High (>45%) (*n* = 45)	
Degree of periodontitis (severe)	7 (33.3)	31 (68.9)	**0**.**006**^**a**^
*CAL measures*			
Proportion of sites/mouth CAL ≥3 mm (%)	42.03 (3.66)	55.41 (2.39)	<**0**.**001**^**b**^
Proportion of sites/mouth CAL ≥5 mm (%)	8.36 (2.28)	13.01 (2.05)	**0**.**016**^**b**^
Mean CAL (mm)	2.63 (0.13)	2.99 (0.11)	**0**.**006**^**b**^
*PPD measures*			
Proportion of sites/mouth PPD ≥4 mm (%)	10.19 (1.40)	14.90 (1.69)	0.184^*b*^
Proportion of sites/mouth PPD ≥6 mm (%)	0.92 (0.32)	1.15 (0.30)	0.676^*b*^
Mean PPD (mm)	2.22 (0.06)	2.40 (0.06)	0.152^*b*^

CAL, clinical attachment loss; PPD, pocket probing depth. The degree of periodontitis is presented as numbers (percentages). The CAL and PPD measures are presented as percentages (standard errors (SE)) or means (SE). Bold *p*-values indicate significant ^a^*χ*^2^ or ^b^Mann-Whitney tests.

In the high TSAT patients, no difference in the periodontitis severity was observed according to the serum ferritin levels: 68.4% *versus* 69.2% presented severe periodontitis (${\chi }^{2}$, $p=0.954$). Moreover, no significant difference was found in the PPD or CAL measures according to the serum ferritin levels (see Supplementary Table S2). Therefore, the rest of the study centred on the groups with normal and high TSAT levels.

### *Alpha* and *beta* diversity analyses showed no clustering according to TSAT

**Figure 1:**
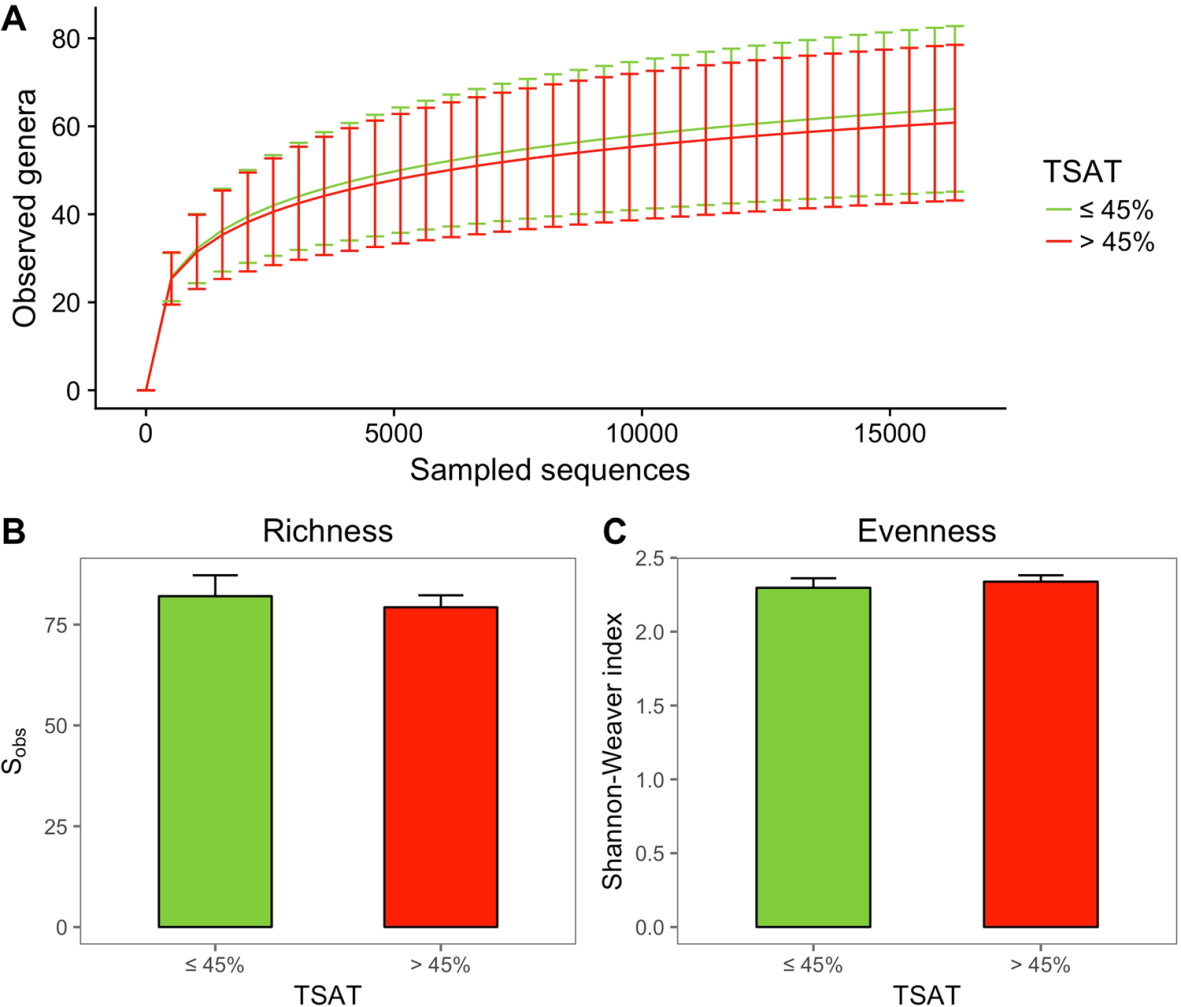
*Alpha* diversity analysis of subgingival microbiota samples. (**A**) Rarefaction curves. The data are presented as the mean ± standard deviation. (**B**) Richness index Sobs. The data are presented as the mean ± standard error. (**C**) Shannon-Weaver evenness index. The data are presented as the mean ± standard error. The results are presented according to the study groups: normal transferrin saturation (TSAT) (≤45%; $n=21$) and high TSAT (>45%; $n=45$). *Alpha* diversity metrics were calculated after subsampling to obtain equal numbers of sequences per library. Mann-Whitney and Student’s t test returned no significant differences in *alpha* diversity measures between the normal and high TSAT samples ($p > 0.05$).

**Figure 2:**
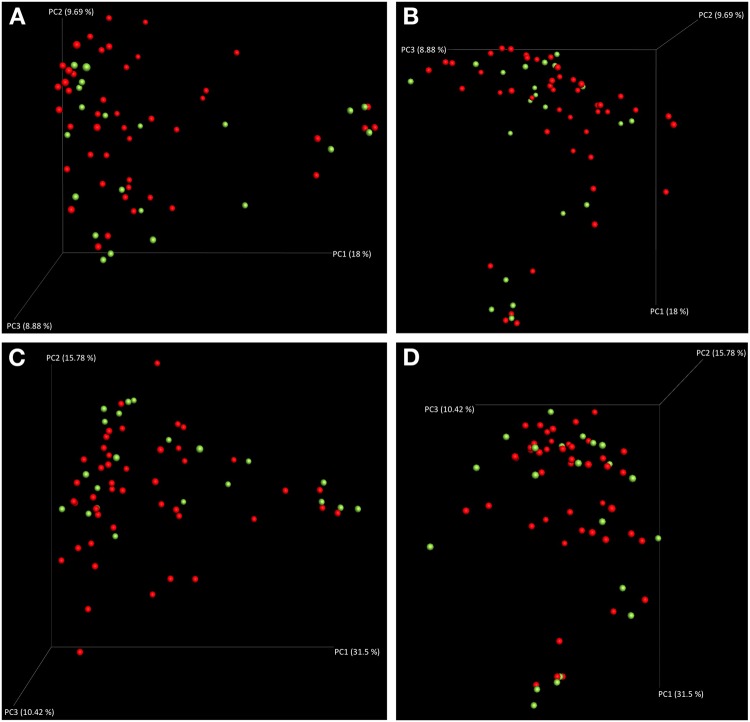
*Beta* diversity analysis illustrating the community structure of the subgingival microbiota. (**A**,**B**) Different views of the 3D PCoA plots calculated with Bray-Curtis metrics. (**C**,**D**) Different views of the 3D PCoA plots calculated with weighted UniFrac metrics. Green, TSAT ≤ 45%; red, TSAT > 45%.

We explored the community structures of the subgingival microbiotas between the TSAT groups using *alpha* and *beta* diversity metrics. The rarefaction curves shown in Fig. 1A reached approximately 60 genus level taxa with 15,000 sampled sequences with no differences between the TSAT groups. No difference was found in the species richness (Sobs) or Shannon-Weaver diversity index according to the TSAT level (Fig. 1B,C). The *beta* diversity analysis did not show any patient clustering according to the TSAT level using the Bray-Curtis (PERMANOVA, $p=0.141$) (Fig. 2A,B) and weighted UniFrac metrics (PERMANOVA, $p=0.153$) (Fig. 2C,D).

### Differences in the relative abundances of specific taxa according to the TSAT level

**Figure 3:**
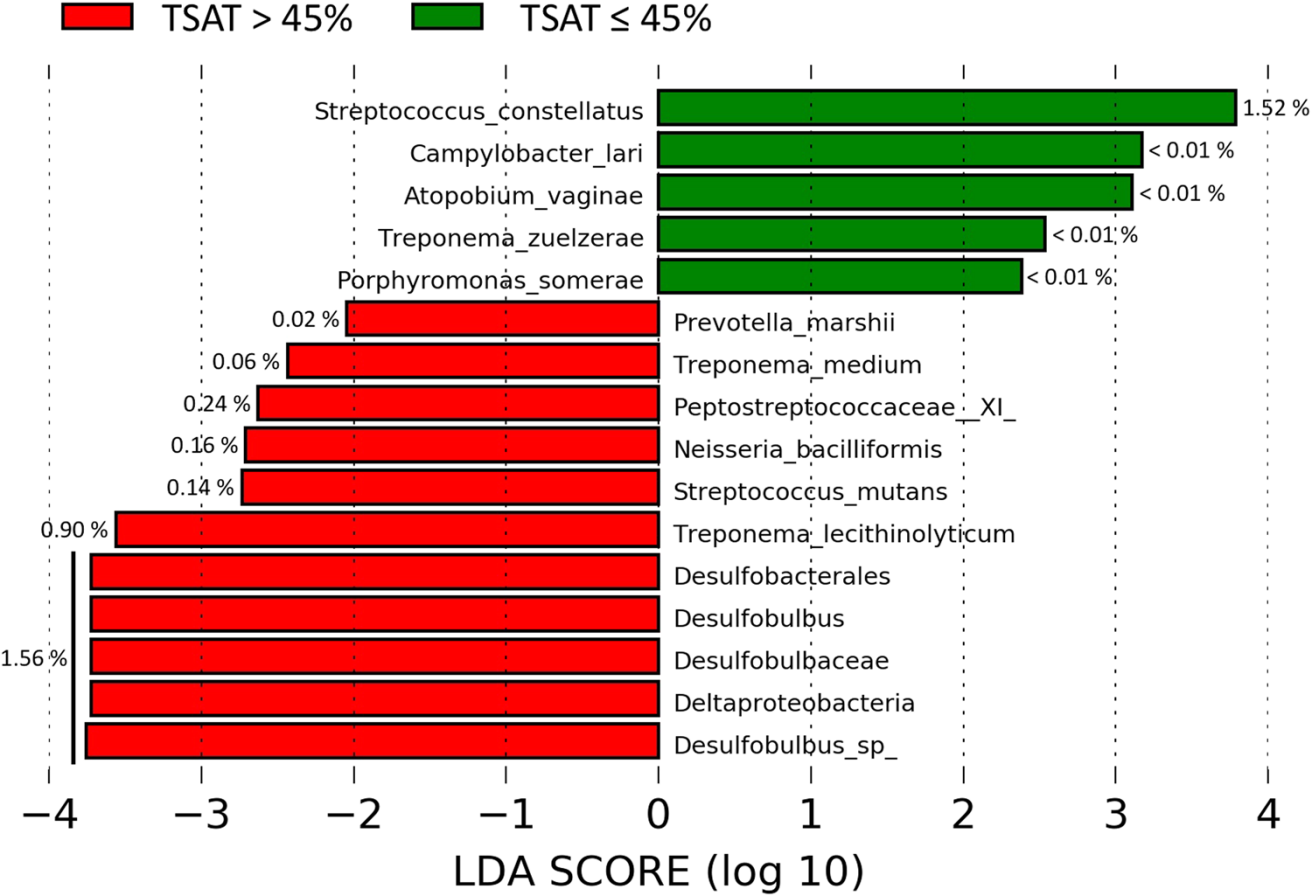
Analysis of taxa relative abundances according to the TSAT level. Taxa showing significant differences in the LEfSe analysis. The percentages are the average relative abundances of the corresponding taxa in the related TSAT group.

We used the linear discriminant analysis with effect size (LEfSe) algorithm to investigate the impact of the TSAT level on the subgingival bacterial composition. In patients with normal TSAT, *Streptococcus constellatus*, *Campylobacter lari*, *Atopobium vaginae*, *Treponema zuelzarae* and *Porphyromonas somerae* were found with significantly elevated proportions in the subgingival microbiota (Fig. 3). *S*. *constellatus* had the strongest linear discriminant analysis (LDA) score.

In patients with high TSAT, the *Desulfobulbus* genus and all taxonomic levels to which it belonged showed increased relative levels and the strongest LDA scores; the unclassified *Desulfobulbus sp*. were also increased. Family *Peptostreptococcaceae [XI]* and the species *Prevotella marshii*, *Treponema medium*, *Neisseria bacilliformis*, *Streptococcus mutans* and *Treponema lecithinolyticum* were also increased in patients with high TSAT. The average relative abundances of taxa with LDA scores <|3| were low (<0.24% of the microbiota).

### Co-occurrence network analysis identified distinct bacterial patterns according to the TSAT level

**Figure 4:**
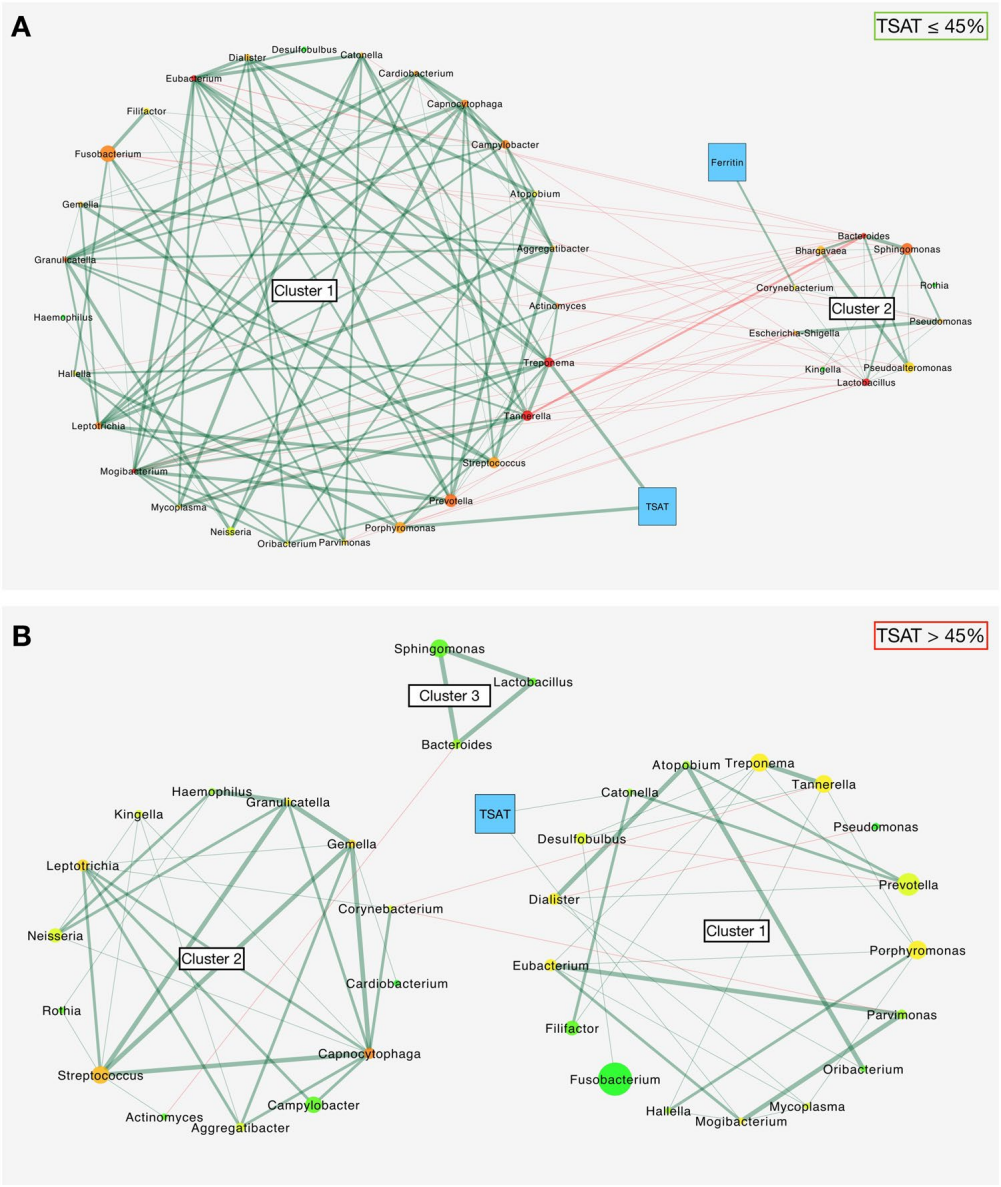
Bacterial co-occurrence patterns with serum iron biomarkers according to the TSAT level. (**A**) Co-occurrence patterns of genera present in at least 70% of patients with normal TSAT ($n=21$); a total of 37 bacterial nodes, TSAT, ferritin and 144 edges are represented. (**B**) Co-occurrence patterns of genera present in at least 70% of patients with high TSAT ($n=45$); a total of 34 bacterial nodes, TSAT and 64 edges are represented. The edges represent 1 (thin line) or 2 or 3 (thick line) significant co-occurrence tests between nodes (green, positive; red, negative). The bacterial node colours represent the number of partners ranging from 1 (light green) to 11 (red); the serum iron biomarkers are shown in blue. The bacterial node sizes represent the mean relative abundance of each taxon.

Co-occurrence tests were performed to investigate interactions among the subgingival core microbiota at the genus level and their relationships with the TSAT and serum ferritin levels. Two co-occurrence network analyses between normal and high TSAT patients were constructed with genera that had prevalence rates of 50% and 70% in the samples. Similar co-occurrence patterns were found within the high and normal TSAT networks with prevalence rates between 50% and 70%. The co-occurrence patterns with a bacterial prevalence rate of 70% are presented in Fig. 4. Thirty-four genera were shared by both patterns. Three genera were found specifically in the pattern from patients with normal TSAT (*Bhargavaea*, *Escherichia*-*Shigella* and *Pseudoalteromonas*). In the samples from patients with normal TSAT, the major genera were *Fusobacterium* (${\rm{relative}}\,{\rm{abundance}}=24.17 \%$), *Prevotella* (12.20%), *Sphingomonas* (6.89%), *Porphyromonas* (6.26%) and *Streptococcus* (6.13%). In the samples from the high TSAT patients, *Fusobacterium* (25.40%), *Prevotella* (12.06%), *Porphyromonas* (7.32%), *Treponema* (6.85%), *Tannerella* (6.31%) and *Streptococcus* (6.15%) were predominant.

In the normal TSAT group (Fig. 4A), the network showed two distinct bacterial clusters. In each cluster, genera were positively and strongly associated with each other (green edges). A clear mutual exclusion was observed between the two clusters (red edges). Classic and putative periopathogens, including *Porphyromonas*, *Treponema*, *Tannerella* and *Filifactor*, were located in cluster 1, whereas frequently health-associated genera, such as *Rothia* and *Corynebacterium*, were located in cluster 2. Moreover, *Porphyromonas* and *Treponema* presented a positive correlation with TSAT as a continuous variable with three significant similarity measures. The serum ferritin levels were positively linked with *Corynebacterium*.

The high TSAT network (Fig. 4B) had less numerous co-occurrences and very few mutual exclusions (64 edges versus 144 in the normal TSAT group). Genera still gathered in clusters, but no opposition was found. Cluster 1 contained genera usually described in periodontitis, including *Porphyromonas*, *Treponema*, *Tannerella* and *Filifactor*. The TSAT level was positively associated with *Catonella* and *Mogibacterium* but with a lower strength, since only the Bray-Curtis dissimilarity returned significant co-occurrences. Similarity measures returned no association between the serum ferritin levels and the subgingival core microbiota, which explained why ferritin did not appear in the high TSAT network.

### Correlation matrices identified different relationships between the subgingival microbiota and clinical parameters

**Figure 5:**
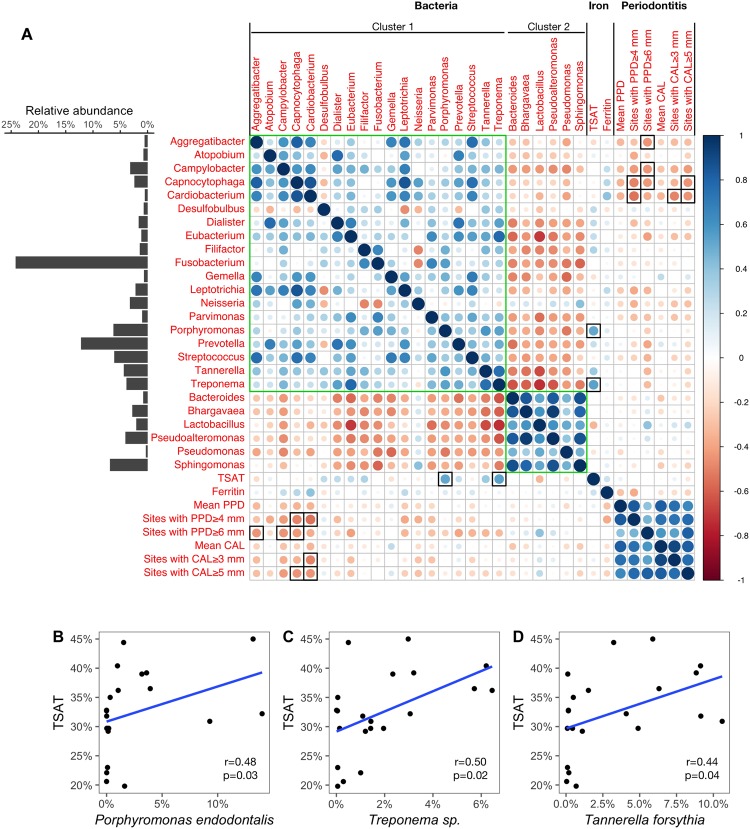
Correlations between microbiota and clinical parameters in patients with normal TSAT (≤45%). (**A**) Correlation matrix among genera found in the co-occurrence network of patients with normal TSAT, serum iron biomarkers and periodontitis measures computed with Spearman’s test. Genera from the co-occurrence network that had an average relative abundance ≥0.5% were used to generate the correlation matrix. Green squares indicate clusters identified in the co-occurrence network. Black squares indicate significant Spearman’s correlations between genera and clinical parameters ($p < 0.05$). The average relative abundances of genera are presented in the bar chart on the left side of the matrix. Spearman’s coefficient values are depicted using the colour gradient scale. (**B**–**D**) Scatterplots of significant correlations between species level taxa and TSAT. Blue lines plot the linear regression slopes.

**Figure 6:**
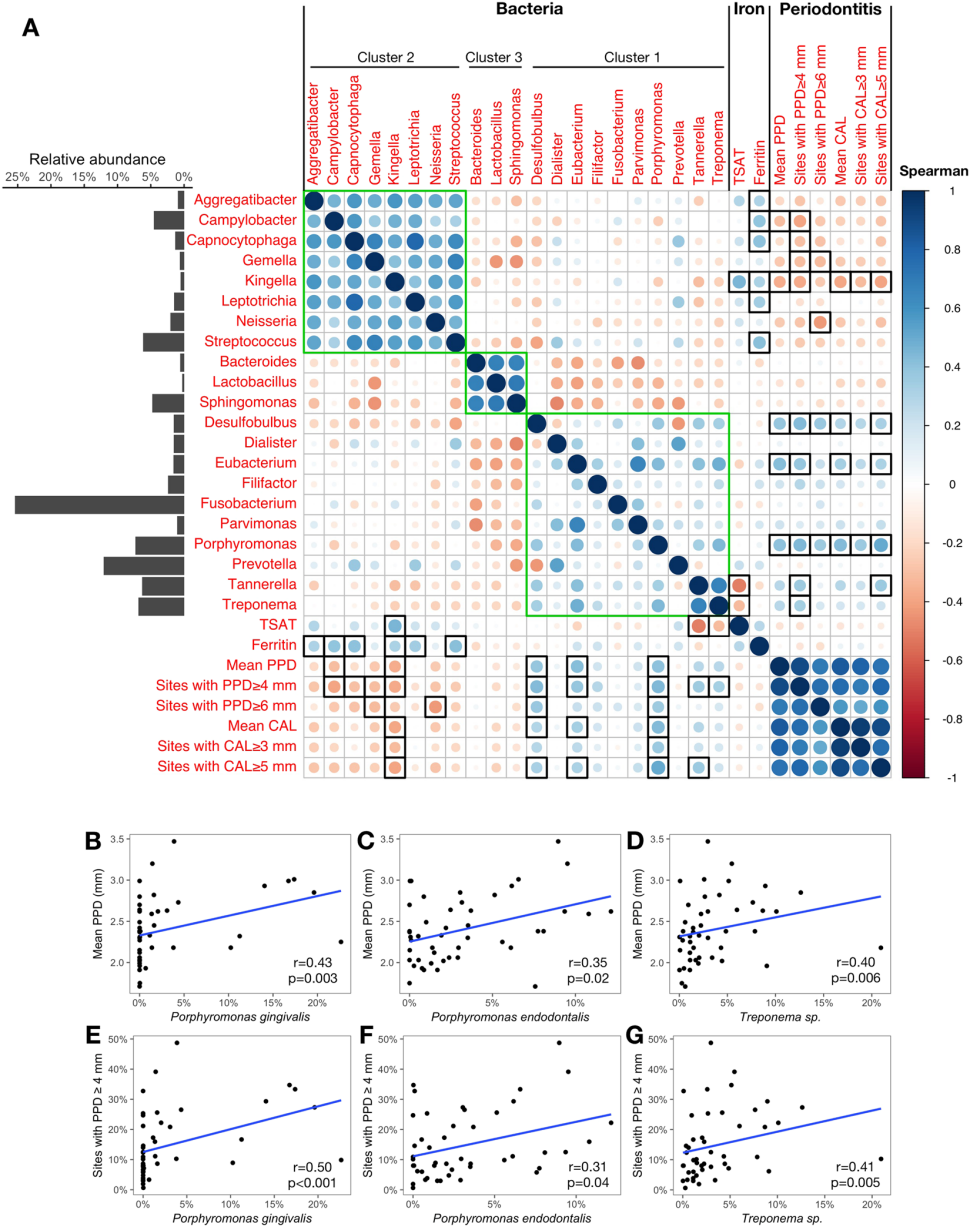
Correlations between microbiota and clinical parameters in patients with high TSAT (>45%). (**A**) Correlation matrix among genera found in the co-occurrence network of patients with high TSAT, serum iron biomarkers and periodontitis measures computed with Spearman’s test. Genera from the co-occurrence network that had an average relative abundance ≥0.5% were used to generate the correlation matrix. Green squares indicate clusters identified in the co-occurrence network. Black squares indicate significant Spearman’s correlations between genera and clinical parameters ($p < 0.05$). The average relative abundances of genera are presented in the bar chart on the left side of the matrix. Spearman’s coefficient values are depicted using the colour gradient scale. (**B**–**G**) Scatterplots of significant correlations between species level taxa and periodontitis measures. Blue lines plot the linear regression slopes.

Associations between bacteria and bioclinical data for both GH and periodontitis were evaluated using Spearman’s correlation matrices. Plots were computed with genera identified in the co-occurrence patterns, TSAT and serum ferritin, and extent/severity measures of periodontitis (Figs 5 and 6).

In patients with normal TSAT, the inter-bacterial correlations showed two clusters (green squares in Fig. 5A). As shown in the co-occurrence bacterial network (Fig. 4A), we observed positive correlations within each cluster and negative correlations between the two clusters.

For the serum iron biomarkers, correlations at the genus level were confirmed between *Porphyromonas* (6.26%) and *Treponema* (3.86%) with the TSAT level ($r=0.52$ and $r=0.54$, respectively; $p < 0.05$). At the species level, *Porphyromonas endodontalis* (2.53%), *Treponema sp*. (1.87%) and *T*. *forsythia* (3.20%) were correlated with the TSAT level ($r=0.48$, $r=0.50$ and $r=0.44$, respectively; $p < 0.05$), as shown in Fig. 5B–D. No significant association was found between the serum ferritin levels and bacteria. For the periodontitis measures, at the genus level *Aggregatibacter* (0.58%), *Campylobacter* (3.17%), *Capnocytophaga* (2.39%) and *Cardiobacterium* (0.51%) presented significant negative correlations with the PPD measures. *Capnocytophaga* and *Cardiobacterium* also presented significant negative correlations with the CAL measures (Fig. 5A).

In patients with high TSAT, very few negative correlations were found between the bacterial clusters (green squares in Fig. 6A), as observed in the co-occurrence bacterial network (Fig. 4B).

With respect to serum iron biomarkers, at the genus level, the TSAT level was positively associated with *Kingella* (0.52%) and negatively associated with *Tannerella* (6.31%) and *Treponema* (6.85%) ($r=0.46$, $r=-\,0.51$ and $r=-\,0.32$, respectively; $p < 0.01$) (Fig. 6A). At the species level, *Campylobacter sp*. (0.93%), *Prevotella sp*. (2.48%) and *T*. *forsythia* (5.52%) were significantly associated with the TSAT level ($r=0.33$, $r=0.31$ and $r=-\,0.46$, respectively; $p < 0.05$). The serum ferritin levels were significantly correlated with *Aggregatibacter* (0.94%), *Campylobacter* (4.56%), *Capnocytophaga* (1.35%), *Kingella* (0.52%), *Leptotrichia* (1.49%) and *Streptococcus* (6.15%) ($r=0.31$, $r=0.39$, $r=0.41$, $r=0.31$, $r=0.34$ and $r=0.41$, respectively; $p < 0.05$) (Fig. 6A). At the species level, *Campylobacter sp*. (0.93%), *Leptotrichia sp*. (0.61%), *Prevotella denticola* (0.99%), *Prevotella intermedia* (0.97%), *Prevotella melaninogenica* (0.55%), *Streptococcus anginosus* (0.68%) and *Streptococcus gordonii* (0.91%) were associated with ferritin ($r=0.31$ to $r=0.37$, $p < 0.05$). Periodontitis measures were positively associated at the genus level with *Eubacterium* (1.58%) and *Desulfobulbus* (1.56%) and negatively associated with *Campylobacter* (4.56%), *Capnocytophaga* (1.35%), *Gemella* (0.62%), *Kingella* (0.52%) and *Neisseria* (2.04%) (Fig. 6A). These genera were low-abundant taxa except for *Campylobacter*. The highly abundant (7.32%) and prevalent (detected in 97.8% of patients with high TSAT) *Porphyromonas* genus was significantly correlated with all periodontitis measures. The two highest coefficients were obtained for the proportion of sites with CAL ≥ 5 mm ($r=0.51$, $p < 0.001$) and the proportion of sites with PPD ≥ 4 mm ($r=0.44$, $p=0.002$). Significant correlations were also found between the proportion of sites with PPD ≥ 4 mm and *Treponema* and *Tannerella* ($r=0.33$ and $r=0.36$, respectively; $p < 0.05$). At the species level, *P*. *gingivalis* (3.08%), *P*. *endodontalis* 3.30%) and *Treponema sp*. (3.61%) were significantly correlated with the mean PPD ($r=0.43$, $r=0.35$ and $r=0.40$, respectively; $p < 0.05$) (Fig. 6B–D) and the proportion of sites with PPD ≥ 4 mm ($r=0.50$, $r=0.31$ and $r=0.41$, respectively; $p < 0.05$) (Fig. 6E–G).

## Discussion

Periodontitis is associated with an inflammatory process related to modifications of the subgingival biofilm^4^. In normal subjects, inflammation leads to iron sequestration within macrophages, which deprives bacteria of iron^25^. In GH patients with abnormally high TSAT, iron bioavailability is increased in biological fluids, including those of the oral cavity, resulting in an increased risk of severe periodontitis^8^. Additionally, evidence of the presence of iron deposits in oral tissues of haemochromatosis patients can be found in the literature^26–30^. Currently, most patients with haemochromatosis are pauci-symptomatic, and the skin and mucosal pigmentation related to iron deposits has become exceptional. However, the presence of asymptomatic iron deposits in oral tissues cannot be excluded.

In the present study, 66 GH patients were sampled to analyse their subgingival microbiotas. As expected, an increased risk of severe periodontitis was observed in patients with a TSAT level >45% together with a significant increase in CAL measures and higher but not significant PPD measures (Table 1). In contrast, no differences in the periodontal status were observed in relation to the serum ferritin level, which could be explained by the physiological role of both markers. Indeed, TSAT provides an insight into iron bioavailability in the plasma, whereas serum ferritin quantifies the total body iron stores. Notably, increased TSAT is associated with the occurrence of NTBI, which is considered a main factor of both iron accumulation and iron-related organ damage in GH^11–13^. Therefore, TSAT was considered the only relevant iron-related biomarker in the present study.

First, the *alpha* and *beta* diversity analyses did not show any significant differences between patients with normal and high TSAT regardless of the metric used. This result could be explained by the fact that the samples had similar origins; the sampling site and host disease status were the same for all samples (subgingival and periodontitis/GH, respectively). As noted in a previous meta-analysis, *beta* diversity allows clustering of samples from different sampling sites and to a lesser extent from opposite conditions (i.e., healthy versus periodontitis)^31^. Despite sharing a high degree of similarity at the community level, significant differences were detected when relative abundance comparisons were conducted between the normal and high TSAT groups using the LEfSe algorithm. *S*. *constellatus*, which was more abundant in patients with normal TSAT, has been isolated from a wide range of sites and infections and is considered a commensal component of the oral microbiota^32^, although it is part of the orange complex according to the Socransky classification^1^. In patients with high TSAT, the increased taxa with major LDA scores were *Desulfobulbus* and *Desulfobulbus sp*. Although not known as a periopathogen, this genus deserves more attention; for instance, Camelo-Castillo *et al*. showed an association between *Desulfobulbus* and the periodontitis severity^33,34^. The increased taxa also included *T*. *lecithinolyticum*, which was associated with periodontitis^35,36^ and was detected in endodontic lesions^37^. Because the periodontal status is led by TSAT^8^, the results from the LEfSe analysis can also reflect the periodontal disease severity. Consequently, we investigated the structures of the microbial communities and their relationships with serum iron biomarkers according to the host iron bioavailability status.

Co-occurrence network comparisons between patients with normal and high TSAT were initially realized at bacterial prevalence rates of 50% (corresponding to the minimal fraction required to compute the core microbiome in the QIIME pipeline) and 70%. In both the normal and high TSAT patients, very similar patterns were found regardless of the prevalence cut-offs used. The number of genera was reduced in the 70% bacterial prevalence patterns, but this discrepancy concerned only low abundance genera, and the 70% cut-off was chosen for network representation. Most of the represented genera, including the main potential periopathogens, were present in the patterns of both the normal and high TSAT patients. This observation remained consistent with the findings of the core microbiota analyses, since all samples originated from patients with similar conditions and from similar sampling sites (i.e., subgingival samples from periodontitis patients). However, the inter-relationships between the different bacterial clusters and the serum iron biomarkers differed according to the TSAT level (Fig. 4). Therefore, the co-occurrence network analysis indicated a shift in bacterial dynamics when the TSAT exceeded 45%, suggesting a change in host-microbiota dynamics when the iron bioavailability was increased.

In patients with normal TSAT, two bacterial clusters were found to compete. Cluster 1 included *Porphyromonas* and *Treponema*, which are well known to contain periopathogenic species^1^, whereas cluster 2 included genera implicated in periodontal health, such as *Rothia* and *Corynebacterium*^38,39^. Moreover, all association measures revealed significant although moderate positive relationships between the relative proportions of *Porphyromonas* and *Treponema* and the TSAT level in both the co-occurrence network and the correlation matrix (Figs 4A and 5A). In addition, the relative abundances of *P*. *endodontalis* and *T*. *forsythia*, which are two species also described as periopathogenic^40^, as well as *Treponema sp.* were the only species associated with the TSAT level.

In patients with high TSAT, the co-occurrence pattern was characterized by a lack of mutual exclusion between clusters and presented less numerous relationships than that of the normal TSAT patients. Neither the TSAT nor the serum ferritin level showed correlations with any genera, since only the Bray-Curtis dissimilarity returned significant results. This finding may raise questions concerning the significance of the correlations found in the matrix between the serum iron biomarkers and several genera. The correlation matrix showed that the clusters seemed to have an opposite impact on periodontal disease. Genera that contained major and putative periopathogens species, such as *Porphyromonas*, *Tannerella*, *Treponema*, *Prevotella*, *Eubacterium*, *Desulfobulbus* and *Filifactor*^1,33,41^, constituted cluster 1 and were positively associated with periodontitis measurements. This result is in accordance with previous studies on the subgingival microbiota in periodontitis patients^31,42,43^ and suggests the presence of a disease-associated dysbiotic microbiota in patients with high TSAT. Conversely, taxa from cluster 2 were negatively associated with the periodontitis measures.

Transferrin is a major player in iron homeostasis^44^. Below 45% saturation, transferrin allows adequate iron delivery to cells. However, bacteria can also access this primary source of bioavailable iron in the host. Indeed, Goulet *et al*. detected the presence of transferrin fragments in subgingival samples from patients with periodontitis, suggesting bacterial or host-related degradation^45^. In addition, an increase in the transferrin concentration in the gingival crevicular fluid was observed in patients with periodontitis^46^. The significant correlations between *Porphyromonas*, *Treponema*, and TSAT in patients with TSAT ≤ 45% support previous studies showing the ability of *Porphyromonas* and *Treponema* species to acquire iron from transferrin^45,47–52^. Moreover, iron provided by *Porphyromonas* through transferrin degradation by gingipains^49^ could supply the biofilm and other periopathogens with iron. Similar nutritional cooperation has been observed between *Treponema* species and *P*. *gingivalis*^53^. These associations between *Porphyromonas*, *Treponema* and TSAT could explain the occurrence of periodontitis in all of these patients.

Increased TSAT > 45% can lead to the formation of abnormal forms of iron in the plasma, such as NTBI, which is considered the key factor of iron toxicity and organ damage in GH^11^. NTBI is a highly bioavailable form of iron, and its uptake by bacteria can benefit the entire microbial community, as suggested by a bacterial co-occurrence network with no mutual exclusion. These data could explain the severity of the periodontitis observed in these patients.

As a whole, this study supports the association between the iron burden, especially elevated transferrin saturation, and the periodontitis severity^8^. Elevation of circulating transferrin-bound iron could facilitate dysbiosis through favouring keystone pathogens, such as *Porphyromonas* and *Treponema*^54^. Once TSAT is high and NTBI occurs, iron acquisition is no longer a challenge. The whole dysbiotic microbiota of these patients is in favour of severe periodontitis, and correlations between periodontal measures and classical periopathogens have been found.

The limitations of this study include its relatively small sample size, although clinical GH related to C282Y homozygosity remains a rare disease^55^. In addition, this study is a case series with no control group available to compare the microbiota of non-GH iron-overloaded patients with normal and high TSAT. Whether the TSAT increase itself or the presence of NTBI in the gingival crevicular fluid plays a role in the occurrence of dysbiosis should be explored. In the gingival sulcus or pocket formed by periodontitis, bacteria are constantly bathed in the gingival crevicular fluid formed by the serum exudate or blood during inflammatory periods. Therefore, iron overload related to GH and variations in TSAT are probably locally reflected in the ecological niche inhabited by the subgingival microbiota.

Moreover, NTBI and labile plasma iron – its highly reactive component – contribute to the production of reactive oxygen species through the Fenton reaction^12,56^. This reaction may take place in the liver, pancreas, bones and heart and participates in the lesions observed in GH^57^. As indicated earlier, mucosal pigmentation in the oral cavity similar to that observed on the skin has also been reported in GH patients, suggesting a local impact of the disease^26^. Cellular NTBI-related toxicity in oral tissues cannot be excluded and may participate in an inflammatory state beneficial to periopathogens^3^ and to a dysbiotic community. Regardless of whether it acts directly by providing iron to bacteria, indirectly via its pro-inflammatory effect or through both mechanisms, future studies should include measurement of NTBI in the serum and gingival crevicular fluid.

In addition, we cannot rule out the impact of a lower iron content in macrophages from GH patients due to hepcidin deficiency. Ferroportin (the cellular iron exporter protein regulated by hepcidin) is overexpressed on macrophage membranes and thus favours an iron-poor phenotype^58^. This macrophage phenotype may alter its innate immunity function and contribute to microbiota dysbiosis. However, the concomitant inflammatory process localized in periodontal tissues could modify this picture. Finally, hepcidin is reported to have direct though small antibacterial activity^59^, and we cannot exclude that a low hepcidin level may directly modulate the oral microbiota.

In this study, we explored associations between the subgingival microbiota, periodontal status and serum iron biomarkers in GH patients with periodontitis. We showed changes in the periodontal status and microbial dynamics according to iron bioavailability as reflected by TSAT. Our data demonstrate that increased TSAT is associated with oral dysbiosis characterized by elevated proportions of periopathogens, which may participate in periodontitis severity. This result and the finding of significant correlations between periopathogenic bacteria and the TSAT level in patients with normal TSAT may suggest a shift in the bacterial iron supply from transferrin to NTBI when NTBI occurs. Taken together, these results indicate that iron bioavailability more than the total body iron stores is part of the equilibrium between the host and its microbiota.

## Methods

### Participants

The participants corresponded to a sub-cohort of the 84 GH patients previously studied by Meuric *et al*.^8^. The demographic data (age, gender ratio, body mass index, smoking habits and frequency of dental visits) of this sub-cohort can be found in Supplementary Table S1. This case series study was approved by the local ethics committee (CPP Ouest V - 10/02-744). The informed consent for study participation has been obtained from all patients and all research was performed in accordance with relevant guidelines and regulations. These subjects were recruited in the Hepatology Department of the University Hospital of Rennes based on C282Y homozygosity and benefited from a full-mouth periodontal examination, including PPD, CAL, gingival bleeding index, gingival index, plaque score and evaluation of periodontitis severity according to the case definitions from the Centers for Disease Control and Prevention and the American Association of Periodontology^60^. The exclusion criteria were pregnancy, presence of another systemic disease, periodontal therapy within the last 12 months and treatment with either systemic antibiotics or drugs known to cause gingival hyperplasia within the last 3 months. Two biomarkers of iron metabolism (the serum ferritin and TSAT levels) were collected at the time of the dental examination together with the clinical data. The 66 patients for whom frozen gingival fluid was available were the sample participants of the present study.

### Sample Collection

For each subject, sterile endodontic paper points (Henry Schein, France) were inserted into the deepest periodontal pocket for 30 seconds after supra-gingival plaque removal. The material was transferred to a sterile tube with 100 *μ*L of sterile distilled water and kept at 4 °C overnight before DNA extraction.

### DNA extraction and sequencing

DNA extraction from the supernatants was performed using the QIAamp DNA Mini Kit (Qiagen, France) according to the manufacturer’s recommendations. The DNA was kept frozen at −80 °C prior to amplification. The V3–V4 regions of the 16S rRNA gene were amplified with the primers 338F (5′-ACTCCTACGGGAGGCAGCAG-3′)^61^ and 802R (5′-TACNVGGGTATCTAATCC-3′)^62^ using 25 amplification cycles with an annealing temperature of 45 °C. The PCR products were sequenced with the Illumina MiSeq at the Get-PlaGe facility (Toulouse, France)^31^.

### Microbiological analysis

Taxonomy at the genus level was assigned using the “Visualization and Analysis of Microbial Population Structures” (VAMPS) analysis pipeline web tool^63^. Default parameters were used for assignment at the genus level with the “Ribosomal Database Project” (RDP release 11) classification^64^. Through the VAMPS process, the best taxonomic hit was assigned for each read. Reads identified as Archaea, Eukarya, Organelle and unknown were excluded from the analysis. *Alpha* rarefaction and diversity were evaluated using the Observed Species metric (Sobs) and the Shannon-Weaver index^65^. *Beta* diversity was calculated using Bray-Curtis^66^ and UniFrac distances^67^. Genera were removed if their relative abundances did not reach 1% in at least one sample or if their mean relative abundances did not reach 0.5% in at least one group (normal or high TSAT) to avoid significant statistical changes with no biological relevance. Genera that had significant differences in presence/relative abundance between healthy and periodontitis subjects in previous studies^33,68–70^ were marked as genera of interest for a species-level taxonomic assignment. This assignment was made using the “Quantitative Insights Into Microbial Ecology” (QIIME version 1.9.1) package software^71^ with a curated database constructed with the “Human Oral Microbiome Database” (HOMD version 14.51)^72^ and full-length 16S rRNA gene sequences of all species belonging to the genera of interest present in the RDP.

### Statistics

The data were analysed using R (version 3.3.3)^73^ with the RStudio software^74^. Statistical tests were chosen following Shapiro-Wilk normality test results for the data distribution and were considered significant for $p < 0.05$. Demographic data, periodontal cases and the extent and severity measures of periodontitis were compared using the ${\chi }^{2}$ and Mann-Whitney tests. The observed richness was analysed using the Mann-Whitney test, and the Shannon-Weaver index was analysed using Student’s t test. The 3D principal coordinates analysis (PCoA) plots were generated using Emperor (version 0.9.51)^75^. The PERMANOVA test was performed to compare *beta* diversity metrics. LDA was computed using the LEfSe algorithm in Galaxy (http://huttenhower.sph.harvard.edu/galaxy/) with the default parameters^76^. Assessments of significant patterns of microbial co-occurrence or mutual exclusion at the genus level of the core microbiota (prevalence ≥ 50%) were performed using Cytoscape (version 3.2.1)^77^ with the CoNet plugin^78^. Only genera found in at least 70% of the normal TSAT or in 70% of the high TSAT samples were represented. Four similarity measures were calculated: the Bray Curtis and Kullback-Leibler non-parametric dissimilarity indices and the Pearson and Spearman rank correlations. The threshold for the similarity measures was set at 0.5, and only edges with merged *p*-*values* < 0.05 (Benjamini-Hochberg correction) were kept. Genera from the co-occurrence networks that had an average relative abundance ≥0.5% were used to generate correlation matrices (calculated using Spearman’s test and plotted using the ‘corrplot’ package for R^79^) between the genera relative abundance and bioclinical parameters with both excess iron and periodontitis treated as continuous variables. Additional correlations between species level taxa and clinical data were also computed with Spearman’s test.

### Ethics approval and consent to participate

A case-series study was conducted after approval by the local ethics committee (CPP Ouest V - 10/02-744). All participants were recruited either during phlebotomy therapy or at diagnosis in the unit of Hepatology, University Hospital, Rennes, between June 2011 and June 2012. The patients gave informed written consent for the dental examination and subgingival sampling. They were instructed on the prevention and treatment of periodontitis as well as on oral hygiene procedures. All research methods were performed in accordance with the relevant guidelines and regulations.

## Electronic supplementary material

Supplementary Tables S1 and S2

Supplementary Table S3

Supplementary Table S4

## Data Availability

The sequence data were submitted to the NCBI Sequence Read Archive (https://www.ncbi.nlm.nih.gov/Traces/study/) under BioProject accession number PRJNA416501. Due to the low sequence counts, the Hemoparo37 and Hemoparo43 samples were withdrawn from the study. The metadata and datasets (OTU tables) supporting the conclusions of this article are included within the article as additional files (see Supplementary Tables S3 and S4, respectively).
