# Supplementary material for: Increased transferrin saturation is associated with subgingival microbiota dysbiosis and severe periodontitis in genetic haemochromatosis
Source: Sci Rep. 2018 Oct 19;8:15532. doi: 10.1038/s41598-018-33813-0 (PMC6195524; doi:10.1038/s41598-018-33813-0)
Supplement: Supplementary file 1 — Supplementary Tables S1 and S2 [file 41598_2018_33813_MOESM1_ESM.pdf]

Increased transferrin saturation is associated to subgingival microbiota dysbiosis and severe periodontitis in genetic hemochromatosis

Emile Boyer, Sandrine Le Gall-David, Bénédicte Martin, Shao Bing Fong, Olivier Loréal, Yves Deugnier, Martine Bonnaure-Mallet, and Vincent Meuric

Table S1: Distribution of the general characteristics of the sample population in accordance with their TSAT level.

Demographics	Total n=66, 100%	Transferrin saturation		*p*-value
		Normal (≤ 45%) (n=21)	High (> 45%) (n=45)	
Gender ratio				
Male	39 (59.1)	14 (66.7)	25 (55.6)	0.393^{a}
Female	27 (40.9)	7 (33.3)	20 (44.4)	
Age (years)	51.3 ± 8.01	52.0 ± 8.02	51.0 ± 8.08	0.640^{b}
Body mass index (kg/m^2)	25.7 ± 3.05	26.3 ± 3.40	25.3 ± 2.86	0.549^{b}
Smoking habits				
Non-smoker	24 (36.4)	9 (42.9)	15 (33.3)	0.454^{a}
Current of former smoker	42 (63.6)	12 (57.1)	30 (66.7)	
Frequency of dental visits				
< 1/years	12 (18.5)	2 (9.5)	10 (22.7)	0.200^{a}
≥ 1/years	53 (81.5)	19 (90.5)	34 (77.3)	

The demographics data are presented as numbers (percentages) or as means ± standard deviations. The *p*-values indicate the results of $^{a}\chi^2$ or ^{b}Mann-Whitney tests.

Table S2: Periodontitis measures of patients with high TSAT (> 45%), in accordance to their serum ferritin level.

Measure of periodontitis	Serum ferritin		*p*-value
	Normal (≤50 µg/L) (n=19)	High (>50 µg/L) (n=26)	
Degree of periodontitis (severe)	13 (68.4)	18 (69.2)	0.954[a]
CAL measures			
Proportion of sites/mouth CAL ≥3 mm (%)	53.74 (3.87)	56.63 (3.05)	0.462[b]
Proportion of sites/mouth CAL ≥5 mm (%)	12.00 (2.10)	13.75 (3.23)	0.863[b]
Mean CAL (mm)	2.92 (0.13)	3.04 ()	0.476[b]
PPD measures			
Proportion of sites/mouth PPD ≥4 mm (%)	15.04 (2.62)	14.80 (2.25)	0.899[b]
Proportion of sites/mouth PPD ≥6 mm (%)	1.18 (0.38)	1.13 (0.45)	0.843[b]
Mean PPD (mm)	2.38 (0.09)	2.42 (0.08)	0.696[b]

CAL, clinical attachment loss; PPD, pocket probing depth. The degree of periodontitis is presented as numbers (percentages). The CAL and PPD measures are presented as percentages (standard errors (SE)) or means (SE). The *p*-values indicate the results of [a]χ^2 or [b]Mann-Whitney tests.
